# The relation between barrier-free environment perception and campus commuting satisfaction

**DOI:** 10.3389/fpubh.2023.1294360

**Published:** 2023-12-06

**Authors:** Jiang Li, Chuyu Zhang, Xiaoxi Cai, You Peng, Shaobo Liu, Wenbo Lai, Yating Chang, Yudan Liu, Liang Yu

**Affiliations:** ^1^School of Architecture and Art, Central South University, Changsha, Hunan, China; ^2^School of Art and Design, Hunan First Normal University, Changsha, Hunan, China; ^3^Human Settlements Research Center, Central South University, Changsha, Hunan, China; ^4^Department of the Built Environment, Eindhoven University of Technology, Eindhoven, Netherlands; ^5^School of Architecture, South China University of Technology, Guangzhou, Guangdong, China; ^6^Hunan Provincial Key Laboratory of Low Carbon Healthy Buildings, Changsha, Hunan, China; ^7^Department of Research and Development Office, Hunan First Normal University, Changsha, Hunan, China

**Keywords:** commuting satisfaction, barrier-free environment, positive emotions, university campus commuting, COVID-19, structural equation modelling

## Abstract

**Introduction:**

The COVID-19 pandemic, which began in the last quarter of 2019, has had a significant impact on urban transportation. With increasing demand for urban transport, the internal roads and public spaces of university campuses play an important role in facilitating commuting and communication between various functional zones. While considerable research has been conducted on route planning, pedestrian-vehicle segregation, and safety management in the internal transportation environment of university campuses, empirical investigations exploring barrier-free inclusive campus environment design and the subjective evaluation of road and public space users in the aftermath of the COVID-19 pandemic are lacking. Recent developments in travel behavior models and positive psychology have led to an increased focus on the correlation among subjective perceptions, attitudes, emotions, and commuting satisfaction in urban transportation and planning design.

**Methods:**

To elucidate this relationship, a study was conducted on the new campus of Central South University in Changsha, Hunan Province, China. Using 312 valid samples, a structural equation model was constructed to analyse the relationship between commuting satisfaction and the barrier-free environment perception of university students regarding the internal transportation environment of the campus.

**Results:**

The results revealed that individuals' instantaneous barrier-free environment perceptions and long-term established positive emotions had a significant positive effect on commuting satisfaction. Furthermore, positive emotions were found to mediate the relationship between commuting attitudes induced by COVID-19, barrier-free environment perceptions, and commuting satisfaction.

**Discussion:**

The results of this study provide a theoretical basis for the necessity of accessibility design in the post-COVID era. In addition, this study considers the perspective of users to provide ideas for the planning and construction of barrier-free campus environments that are based on convenient and inclusive design.

## 1 Introduction

In recent years, interest in the study of mental health and subjective wellbeing has grown (1–3). Consequently, studies on commuting satisfaction have gained attention in the fields of positive psychology and environmentally sustainable development (4). Commuting satisfaction is a unique area within the realm of subjective wellbeing (1, 5); it primarily focuses on factors such as commuting enjoyment (6), commuting stress (7, 8), customer satisfaction with public transport services and passenger perceptions of public transit quality (9), and satisfaction with public transportation and other modes of transport (10, 11). By exploring the relationship between commuting satisfaction and social life participation, the influence of commuting satisfaction on subjective wellbeing can be examined (12). However, the spread of the coronavirus (COVID-19) has exerted a significant influence on public transport travel behaviour (13–16). During severe pandemic lockdowns, university campuses suspended commuting; consequently, college students' mental health has become a prominent focus of research in the post-COVID-19 pandemic era (17–19). Commuting satisfaction is believed to have a positive impact on college students' participation in campus activities and academic success as well as to be beneficial for emotional health (20). Therefore, research on commuting satisfaction can help improve the post-pandemic subjective wellbeing of college students and alleviate their mental health problems.

The construction of barrier-free environments has been demonstrated to play a vital role in improving the quality of cities and the happiness of residents (21). The national “14th Five-Year Plan” in China has explicitly proposed accelerating the improvement of barrier-free engineering and the construction design standard system. Moreover, the Ministry of Education has also stated that the construction of barrier-free campus environments should be promoted at all levels and types of schools (22). The implementation of barrier-free humanised construction in campus spaces should focus on user needs and the current situation. To achieve this, it is important to consider transportation systems and landscape environments. A good campus transportation system is a vital link in realising barrier-free access. Pedestrian–vehicle diversion measures play a variety of roles in ensuring smooth traffic flow and promoting the formation of central pedestrian zones in the campus landscape (23). In addition, green space accessibility within a certain buffer zone has a positive impact on the mental health of college students (24), with the ease of accessibility to green spaces directly affecting college students' perception of the use of the space (25). Barrier-free environments stimulate commuting behaviour by providing user-friendly spaces with high walkability and accessibility (26, 27). Few studies have investigated commuting satisfaction from the perspective of the urban environment. Ye and Ta noted that better-built environments and green spaces can provide improved commuting satisfaction (28–30), while Feng and Dong reported that the higher the accessibility to amenities, public transportation, and green spaces, the more satisfying commuters are with their daily commute (31, 32). Although many studies have discussed the link between urban environments and commuting satisfaction, research addressing the mechanisms responsible for the impact of barrier-free environments on commuting satisfaction is lacking. Some studies have indicated that benign coupling of individual wellbeing with the environment can promote the sustainability of individual health (33). Therefore, this study introduces individuals' momentary perceptions of barrier-free environments, which can provide a deeper understanding of the effect of accessibility on the commuting behaviours and subjective wellbeing of college students. Understanding these effects is essential to promote the efficient construction of barrier-free environments, which can enhance the quality of cities and the happiness of residents in the post-COVID-19 period; this study provides valuable insights to achieve this goal.

Commuting satisfaction is influenced by both commuting attributes and individual subjective factors (34). Commuting satisfaction varies significantly depending on the mode, duration, and purpose of the commute (35–38). However, objective factors such as commuting attributes alone are not sufficient to fully capture the influence of the environment and individuals on determining commuting satisfaction. Although direct questions about travel satisfaction yield a mapping of satisfaction with actual travel attributes, satisfaction ratings may also be influenced by subjective factors such as respondents' attitudes, emotions, and psychological expectations at the time of the interview. It has been shown that commuters' long-term attitudes and pre-existing emotions towards commuting behaviour affect commuting satisfaction both directly and indirectly. A person's attitude towards a particular mode of transportation can directly influence their mood while commuting. For example, those who prefer biking as their mode of transportation are more likely to be happy and satisfied with their commute as opposed to those who resort to biking owing to a lack of alternative options. Additionally, attitudes can indirectly affect travel satisfaction by influencing a person's choice of travel mode (39, 40). For example, commuters who support low-carbon and environmentally friendly travel will be more satisfied with commuting via bicycle or walking (41). Although attitudes are often considered control variables for self-selection, several studies have concluded that attitudes do play a significant role in influencing travel behaviour (42). Positive emotions are considered to be a determinant of subjective wellbeing (43). Ettema et al. (44) reported that positive emotions related to travelling have an impact on subjective wellbeing (34, 44). Chatterjee et al. (45) highlighted the strong connexion between commuting and the overall health of individuals, and found that active commuting modes, such as bicycling or walking, can significantly improve emotional wellbeing (45, 46). Particularly in the context of a pandemic, negative passenger attitudes towards public transport and reduced travel mobility as a result of COVID-19 can have a detrimental impact on the wellbeing of individual public transport commuters. This, in turn, affects physical and mental health, which ultimately leads to a decline in commuting satisfaction (47–51). In addition, the concept of psychological expectations has often been overlooked in studies on commuting satisfaction, with few studies exploring the impact of inconsistencies between actual and desired commuting times on commuting satisfaction (38, 41). The literature suggests that individual perceptions of environmental stimuli based on individual differences and characteristics, as well as psychological factors, also contribute to the overall assessment of commuting satisfaction in a given environment (3, 52, 53). Interestingly, previous studies have examined a variety of factors that contribute to an individual's satisfaction with their commute. These factors include their perception of the environment, attributes of their commute, attitudes towards commuting, and overall mood. Since the COVID-19 outbreak, several studies have addressed the impact of psychological factors on commuting behaviour under the influence of COVID-19 (13, 54–56). However, only few studies have examined the interrelation and mutual influence of these various factors in the post-COVID era. Research on the connexion between barrier-free campus environments and college students' commute satisfaction has been limited. Therefore, these are important areas for future investigation.

College students' campus involvement has been largely reduced by COVID-19 (57). Students commuting behaviours were limited in most universities to prevent the potential spread of the epidemic (58). Under this circumstance, some university students experience reduced social activities and other troubles, such as financial stresses and academic frustrations, which may cause negative outcomes regarding emotional and perceived satisfaction (59). One study found that most college students felt the pandemic has had a negative impact on their educational experiences (20). After experiencing the COVID-19 lockdown, College students' wellbeing is influenced to a greater extent by the campus environment, and the blockade associated with COVID-19 provides individuals with a new way to perceive the value of campus spaces (60). Their need for barrier-free inclusive campus environment design and use of road and public space has been amplified, which may also have implications for the relationship between barrier-free environment perception, positive emotions, commuting attitudes, and satisfaction. College student populations have unique commuting needs and behavioural choices. Few studies have examined the commuting behaviour of college students. Most of these studies have focused on the choice of commuting mode to and from campus (1, 61), while a few others have explored college students' satisfaction with their campus commutes (20, 62–64). However, empirical investigations exploring barrier-free inclusive campus environment design and the subjective evaluation of road and public space users are lacking, the mechanism by which a perceived barrier-free campus environment affects commuting satisfaction is unclear; this is the main focus of this study. Assessing the commuting satisfaction of college students requires combining individual demographic characteristics, commuting attitudes to COVID-19, positive emotions towards campus commuting behaviours, and momentary perceptions of the barrier-free campus environment. Based on these factors, this study explores the relationship between commuting attitude and commuting satisfaction, as well as the mediating roles of barrier-free environment perception and positive emotion based on positive psychology theories and a model of travel behaviours. The results of this study can provide theoretical references for humanised planning and construction of barrier-free campus environments.

## 2 Conceptual framework

Subjective wellbeing is a concept that transportation researchers have explored as an alternative to utility when evaluating outcomes for travellers. Several researchers have studied the relation between travel and overall wellbeing. According to Ettema et al. (44) travel affects wellbeing in three ways: it is a means to get to doing activities that impact well-being, the travel experience affects one's experience of the activity that impacts well-being, and the experience of travel in and of itself affects well-being.

The last of these effects – commute wellbeing, also known as commute satisfaction or commute happiness, has been the subject of much research (65). Commute wellbeing relates to the daily commute experience and how it accumulates over time to establish a general level of satisfaction (44). Contributing factors to commute satisfaction include travel distance, time, and cost, as well as travel time variability and many other non-instrumental or “affective” factors that can be referred to as perceived commute quality. Our research focuses in-depth on perceived commute quality.

The concept of barrier-free environment perception involves the psychological relationships between individuals and their surroundings. This theoretical perspective in cultural geography is largely based on self-reported data obtained through interviews or questionnaires, which reflect subjective views of the environment (66, 67). Commuting satisfaction, on the other hand, is a comprehensive evaluation of the transportation service system and the overall commuting experience (45). Research has demonstrated that environmental perceptions can influence satisfaction levels by affecting behavioural intentions. The built environment, particularly its perceived qualities, has a significant impact on walking behaviour. Human behaviour is influenced more by the perceived environment than by the actual environment. Environmental perception focuses on the interactions between individuals and their surroundings in a given environment. Exposure to green spaces while commuting has been found to increase comfort and ultimately improve commuting satisfaction (28). The built environment can indirectly affect commuting satisfaction by influencing commuting behaviours and attributes (68). A barrier-free environment along commuting routes can directly influence individuals' moods and environmental perceptions. Different people may have varying perceptions of the same environment, leading to different satisfaction ratings for the commuting experience.

Emotional responses are the feelings, emotions, etc., that people experience in their daily lives; these comprise a person's emotional wellbeing (46). Emotional responses play a significant role in individuals' overall wellbeing and can affect satisfaction in various aspects of life, including a daily commute. Although objective factors such as commute attributes certainly play a role in satisfaction assessment, subjective factors such as mood and emotions also play significant roles. Research has shown that positive moods can lead to higher levels of satisfaction. Studies in the travel domain have shown that emotions such as enjoyment and stress can affect satisfaction judgments (68). Similarly, factors such as poor weather conditions, bad moods at work, and stressful work conditions can also affect commuter satisfaction assessments (69). However, some scholars have discussed the positive impact of moods on commuting satisfaction (70).

Commuting attitudes, i.e., preferences or opinions held by commuters related to commuting behaviour, may affect commuting satisfaction by influencing commuting behaviour. Travel behaviour theories have acknowledged this connexion (71). Commuters tend to have certain expectations regarding their ideal commute; their satisfaction is based on the difference between these expectations and their actual experience (12). For example, according to Huang et al. (72) perceived satisfaction is used to uncover whether the actual appearance and dedicated urban infrastructure (e.g. well-lit urban trails, water fountains, public stretching and exercise equipment, and signage and wayfinding system) correspond with the aspirations and preferences of users. Previous studies have shown that pre-existing attitudes towards a particular commuting mode can affect satisfaction associated with that commute (73). For example, a study on college students found that those who viewed commuting as enjoyable or useful had higher levels of satisfaction, whereas those who only saw it as a means to an end were less satisfied (10). Handy and Thigpen (62) further suggested that using a preferred commuting method may lead to higher levels of satisfaction. Owing to the COVID-19 outbreak, a significant impact on the use of public transportation is expected as commuters may consider the risk of infection (14). Commuting behaviour may be limited owing to emotional, attitudinal, or other psychological factors, and concerns about COVID-19 may overshadow perceptions of commuting satisfaction. Therefore, this study focuses on understanding attitudes towards campus commuting after the pandemic.

## 3 Hypothetical structure

**Figure 1:**
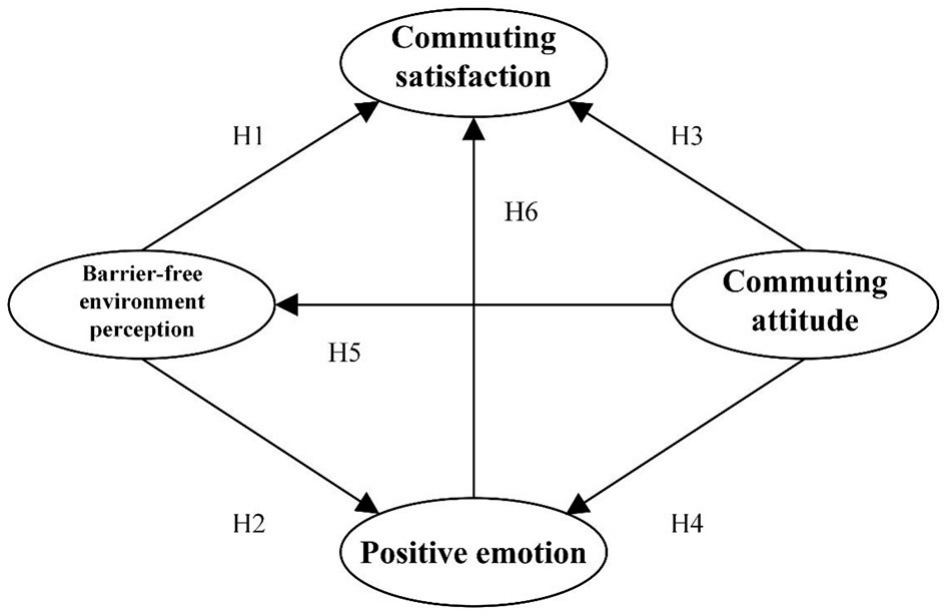
Diagram of the conceptual model.

In this study, we synthesised the results of preliminary research and theoretical models of travel behaviour to construct a conceptual model (see Figure 1). The model proposes a relationship between potential variables and emphasises that an individual's momentary perception of the barrier-free environment may affect their assessment of commuting satisfaction. In addition, long-term attitudes to COVID-19 and positive emotions towards campus commuting behaviours may influence the momentary perception of a barrier-free environment and assessment of commuting satisfaction. Based on this, we formulated eight research hypotheses (from H1 to H8). H1 and H2 considered the relationship between barrier-free environment perception to COVID-19 and commuting behaviour. In public transport, green space exposure was only considered in one study about commute satisfaction (28). Still, its relationship with a barrier-free environment remains to be further verified. H3, H4, H5, and H6 are based on existing literature but will be applied to new data collected after the COVID outbreak, updating the original research in a new context. H7 and H8 considered the mediate effect of positive emotions. Commute satisfaction refers to the satisfaction level of individuals regarding their daily commute to and from campus. This satisfaction can be partially evaluated based on a cognitive assessment of the trip. This definition is in line with the conceptualisation of De Vos and Witlox and builds on the work of Ettema et al. (74) who established cognitive evaluation as a crucial measure of travel satisfaction (5, 74). The attitude towards campus commuting behaviour is a complex multidimensional construct that can be influenced by various factors such as individual characteristics and the environment. However, the measurement of individual attitudes towards campus commuting behaviour is rarely reported in the environmental and planning literature. Emotions, attitudes, or perceived commuting qualities can significantly limit the choice of commuting mode and, therefore, affect satisfaction. Moreover, exploring the correlation between commuter satisfaction and barrier-free environment perception is a critical component of the conceptual model.

## 4 Methodology

### 4.1 Study locations

**Figure 2:**
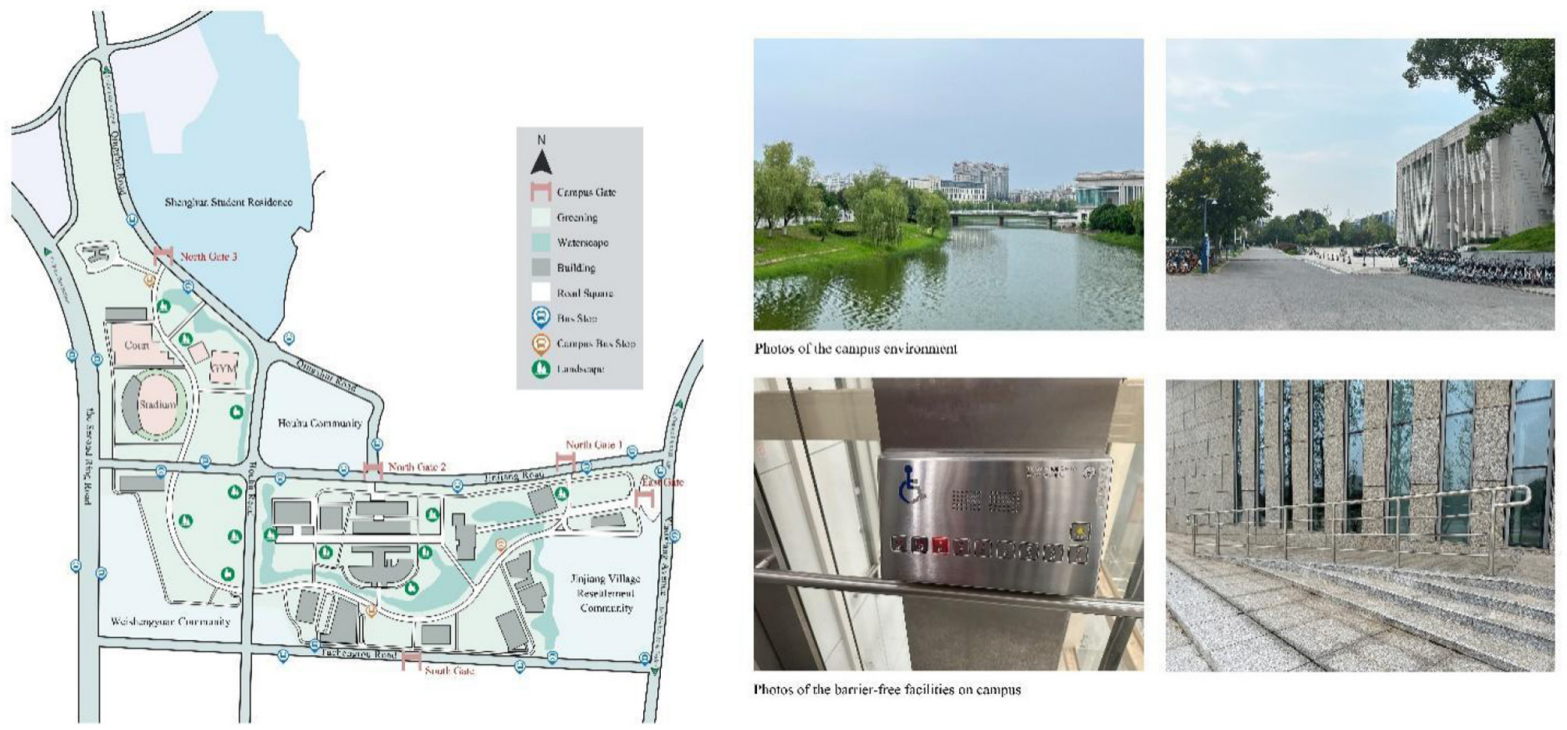
Plan of the study site and photographs of the location.

The new campus of Central South University (CSU) in Changsha, Hunan Province, China is situated in a prime location. It is located north of Tuchengtou Road, south of Jinjiang Road, west of Xiaoxiang Avenue, and east of the city's second ring road. The campus is connected to the main campus of CSU in the north, the Shenghua Student Residence Area of CSU in the northeast, the Xiangjiang River Scenic Belt in the east, the University Town Science and Technology Park in the south, and Yuelu Mountain and Houhu Park in the north. The overall layout of the campus is an L-shape (see Figure 2) covering a vast area of 1.41 km^2^ as of May 2023. The planning area includes 0.83 km^2^ of building areas, including 10 secondary colleges and three secondary teaching and research units. The administrative office of the entire university will also be included. The site has five entrances and exits that allow for convenient transportation via various commuting modes. Additionally, the campus is well-equipped with teaching and public service facilities, providing a conducive environment for students and staff to learn, research, live, and receive services (75).

### 4.2 Questionnaire design

The questionnaire in this study consisted of five sections. The first section collected demographic information such as gender, age, education level, housing, distance from the residential address to the frequented campus area, purpose of commuting, commuting mode, and commute duration. The second section measured respondents' commuting satisfaction using the Satisfaction of Travel Scale (STS) proposed by Ettema et al. (74, 76), which includes six items centred around emotional experience and cognitive appraisal. The third section used the Warwick Edinburgh Positive Mental Scale (C-WEMWBS) (77) to gauge commuters' positive emotions while commuting on campus. The fourth section focused on collecting information about the respondents' commuting attitudes and preferences in the post-COVID-19 pandemic era (20) based on seven questions aimed at understanding their priorities and motivations when commuting on campus. The fifth section utilised the Perceived Barrier-Free Environment Scale, which was adapted from the well-established scale developed by Liu and Cerin (53, 78) to suit the research scenario. This section was used to measure the perceived level of the overall barrier-free environment of the campus. All questions, except for those in the first section, were based on a Likert-type scale ranging from 1 to 5 (where 1 is strongly disagree, 2 is somewhat disagree, 3 is neither disagree nor agree, 4 is somewhat agree, and 5 is strongly agree).

### 4.3 Data collection

In April and May 2023, questionnaire data were collected from university students through random sampling. Throughout the survey period, 350 questionnaires were distributed and 320 were returned. Of these, 312 were deemed valid, resulting in a valid questionnaire recovery rate of 89.14%.

## 5 Results

### 5.1 Descriptive statistics

The statistical results indicate that the respondents were equally distributed in terms of gender: 43.6% were men and 56.4% were women. The majority of the respondents were young, with 78.2% falling within the age range of 17–22 years, followed by 18.6% in the age range of 23–26 years. Regarding education, the majority of the respondents were undergraduates (78.8%), followed by 17.6% with master's degrees and 3.5% with doctoral degrees. The majority of respondents lived in shared quarters (91.6%). Based on these demographic characteristics, the sample was well-represented and could meet the data requirements for further empirical research (see Table 1).

**Table 1:** Demographic characteristics of survey respondents (*n* = 312).

**Variable**	**Category**	**Percentage**
Gender	Male	136 (43.6%)
	Female	176 (56.4%)
Age	17–22 years	244 (78.2%)
	23–26 years	58 (18.6%)
	>26 years	10 (3.2%)
Education	Undergraduate	246 (78.8%)
	Masters	55 (17.6%)
	Doctorate	11 (3.5%)
Housing	Dormitory	285 (91.3%)
	Sharing with others	5 (1.6%)
	Renting a room alone	12 (3.8%)
	Living with family	10 (3.2%)

Table 2 presents data on the behavioural variables of the respondents. The statistics reveal that 40% of the respondents commuted within a distance of 1–2 km. In addition, 72.4% of the respondents commuted to attend classes. More than 50% of the respondents used electric bicycles or motorcycles as their mode of transportation, and over 70% reported a commute time of <15 min.

**Table 2:** Behavioural factors of survey respondents (*n* = 312).

**Variable**	**Category**	**Frequency**
Distance from residence to frequented campus area, x (km)	x < 1	73 (23.4%)
	1 ≤ x < 2	124 (39.7%)
	2 ≤ x < 3	79 (25.3%)
	x ≥ 3	36 (11.5%)
Purpose of this/last campus commute	Attend class	226 (72.4%)
	Self-study	27 (8.7%)
	Exercise	16 (5.1%)
	Leisure	6 (1.9%)
	Work or administrative affairs	30 (9.6%)
	Other	7 (2.2%)
Mode of transportation for this/last on-campus commute	Walking	76 (24.4%)
	Bicycle	53 (17.0%)
	Electric bicycle or motorcycle	157 (50.3%)
	School bus	11 (3.5%)
	Private car	10 (3.2%)
	Taxi or internet taxi	5 (1.6%)
Length of transportation for this/last campus commute (min)	< 15	230 (73.7%)
	15–45	74 (23.7%)
	>45	8 (2.6%)

### 5.2 Commuting attributes, barrier-free environment perception, and commuting satisfaction

**Figure 3:**
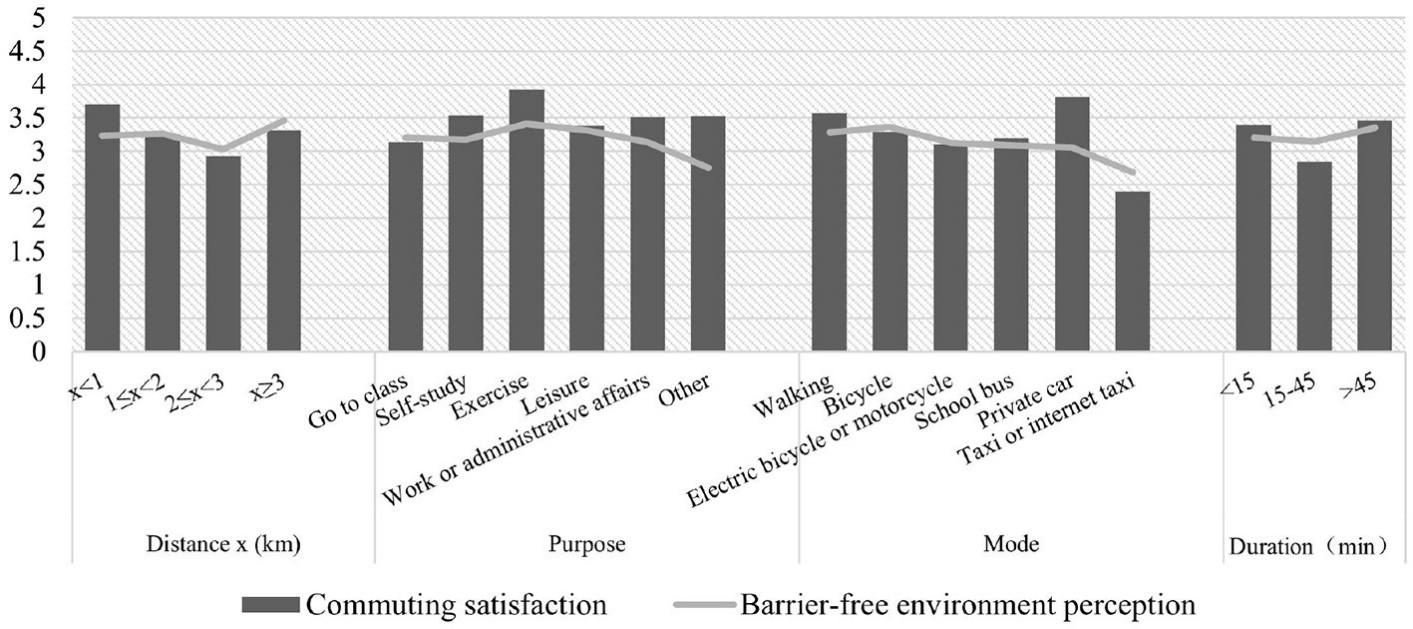
Average levels of barrier-free environment perception and commuting satisfaction for different commuting attributes.

Figure 3 shows variations in the commuting satisfaction and barrier-free environment perception based on different factors, such as the commuting distance, purpose, mode, and duration. The results indicate that the perception levels were influenced by the commuting distance, with the highest perceived level reported for distances ≥3 km. Commuting for sports and leisure resulted in the highest barrier-free environment perception level. Among the different modes of commuting, bicycling yielded the highest perceived level, whereas taking a taxi or internet taxi produced the lowest perception of a barrier-free environment. The commute duration had an average perception level showing a U-shaped pattern, with college students having the lowest perception of a barrier-free environment during a 15–45 min commute. Based on these results, it can be inferred that college students may take different routes during their campus commutes, leading to differing levels of barrier-free environment perception.

Figure 3 shows the average satisfaction levels for different commuting attributes. The results indicate that commutes under 1 km produced the highest satisfaction levels, possibly because of the availability of walking and bicycling as convenient options for short commutes, which provide exercise and allow for campus exploration. The respondents also showed higher satisfaction when commuting actively for exercise and self-study. Private cars, walking, and bicycling were also highly satisfactory modes of transportation, likely because they are predictable, typically used for short commutes, and carry less risk of infection than public transportation. Longer commutes (over 45 min) were found to be the most satisfactory, followed by commutes under 15 min. Medium-to-long commutes showed lower levels of satisfaction, which is consistent with the perceived level of the barrier-free environment. These findings suggest a complex relationship between perceptions of a barrier-free environment and commuting satisfaction, requiring further investigation.

### 5.3 Reliability and validity analysis

A reliability test was conducted on the measurement model based on valid data collected from the questionnaires. The results showed that the Cronbach's alpha values of the four latent variables (commuting satisfaction, barrier-free environment perception, commuting attitude, and positive emotions) ranged from 0.885 to 0.951, indicating strong internal reliability (see Table 3). The overall Cronbach's alpha value of the scale was 0.942 (>0.700), and the Kaiser–Meyer–Olkin (KOM) sampling fitness number was 0.926 (>0.700). The significance of the Bartlett's sphericity test was 0.000 (< 0.001), indicating that the data were suitable for exploratory factor analysis.

**Table 3:** Fitting factors for measurement scale problems.

**Structure variables**	**Code**	**Source of observation indicators**	**Standardised factor loadings**	**Cronbach's alpha value**	**CR**	**AVE**
Commuting satisfaction (CS)	CS01	Time elapsed during this/last campus commute	0.853	0.936	0.929	0.686
	CS02	Fatigue level during this/last campus commute	0.857			
	CS03	Level of boredom during this/the most recent campus commute	0.814			
	CS04	Level of service during this/the most recent campus commute	0.753			
	CS05	Convenience of commuting to campus this time/last time	0.843			
	CS06	Overall efficiency of the commute this time/last time	0.845			
Positive emotion (PE)	PE01	I have always felt optimistic about the future	0.749	0.951	0.946	0.556
	PE02	I have always felt useful	0.785			
	PE03	I have always felt relaxed	0.730			
	PE04	I am always open to making new friends	0.718			
	PE05	I have always had a lot of energy	0.828			
	PE06	I have been able to solve problems well	0.819			
	PE07	I have been able to think clearly	0.802			
	PE08	I am always satisfied with myself	0.815			
	PE09	I always feel good about my relationships with others	0.735			
	PE10	I am always confident	0.751			
	PE11	I have been able to make my own decisions	0.700			
	PE12	I have always felt loved by others	0.634			
	PE13	I am always interested in new things	0.646			
	PE14	I am always in a good mood	0.693			
Barrier-free environment perception (BEP)	BEP01	I can easily find accessible facilities (e.g. blind alleys, accessible elevators, etc.) and accessible services (e.g. Braille signs, audio announcements, etc.) on campus	0.757	0.921	0.916	0.645
	BEP02	I am very satisfied with the lighting conditions in pathways, corridors, and stairways on campus	0.762			
	BEP03	The existence of curbed ramps, blind alleys, barrier-free access, Braille signage, and other barrier-free facilities on campus makes my commute more comfortable and convenient	0.799			
	BEP04	I think the overall accessibility of the campus is very good	0.870			
	BEP05	I think the number of barrier-free facilities and services on campus is sufficient	0.870			
	BEP06	I think the campus has good accessibility to all functional areas	0.750			
Commuting attitude (CA)	CA01	It is important to get to my destination as quickly as possible after the epidemic	0.719	0.885	0.900	0.566
	CA02	Flexibility in commuting time is important after the epidemic	0.832			
	CA03	Predictability of the length of the commute is important after the epidemic	0.786			
	CA04	Getting to my destination by the cheapest mode of transportation is important after the epidemic	0.738			
	CA05	Environmentally friendly travel is important after the epidemic	0.602			
	CA06	Minimising commuting in rain or snow is important after the epidemic	0.789			
	CA07	Minimising commuting in very hot or very cold weather is important after the epidemic	0.777			

Confirmatory factor analysis was then conducted to validate the results. The standardised factor loading values for each question item under each latent variable were greater than the minimum threshold criterion requirement of 0.500, while the construct reliability (CR) values of each latent variable were >0.900 (>0.700). The average variance extracted (AVE) values of each latent variable were also greater than the minimum threshold criterion requirement of 0.500, indicating good reliability and validity of each latent variable in the scale (see Table 3).

To determine the discriminant validity of the latent variables, their correlation coefficients were compared with the square root of the AVE of each latent variable. The results showed that the correlation coefficients for each latent variable with the other latent variables were smaller than the square root of the AVE of each latent variable, indicating good validity of the scale data.

### 5.4 Structural equation model analysis

#### 5.4.1 Structural equation model goodness-of-fit analysis

The model parameters were estimated using the maximum likelihood method. The analysis of the overall goodness-of-fit of the model shows (see Table 4) that all of the indexes met the test criteria. Therefore, the model fit was good, and the results were acceptable.

**Table 4:** Structural equation model fit indices.

**Criterion**	**CFI**	**TLI**	**RMSEA**	**χ^2^/df**
Value	0.930	0.923	0.061	2.149

#### 5.4.2 Hypothesis testing results

**Figure 4:**
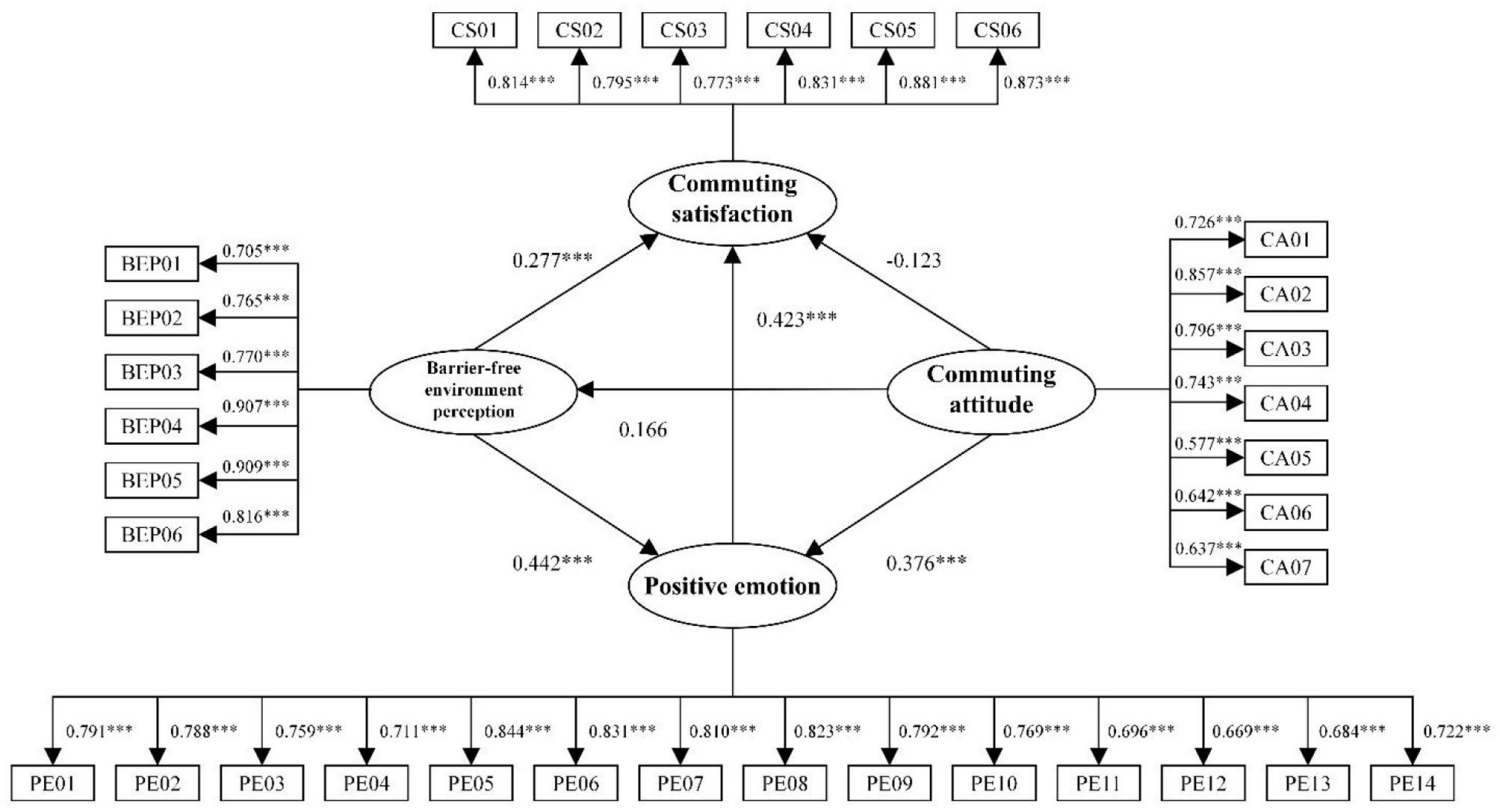
Estimated road map of the standardised parameters of the model. ***p ≤ 0.01.

**Figure 5:**
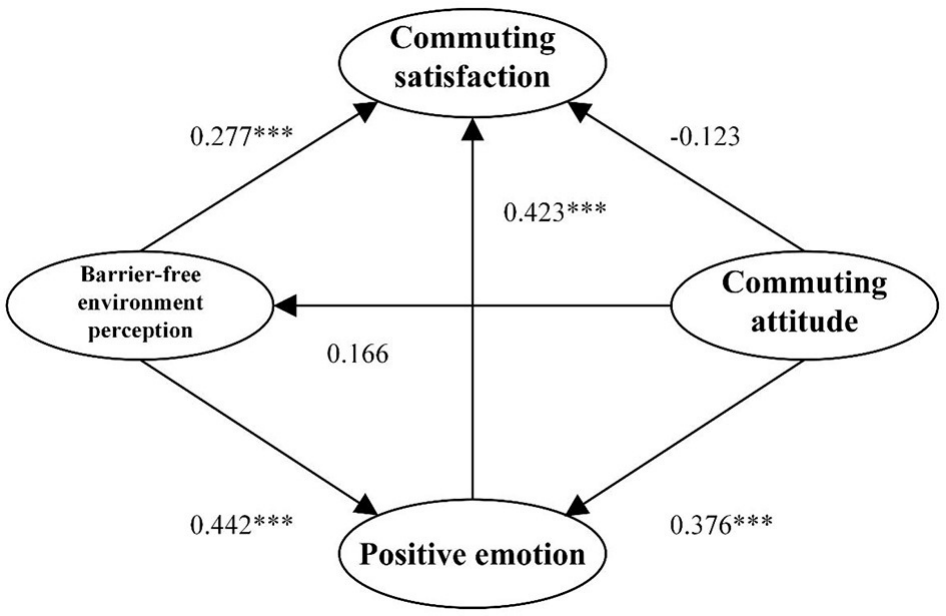
Modified theoretical model. ***p ≤ 0.01.

The test criterion for path analysis was set at a significance level of p < 0.050. We analysed the results of the measurement model (see Table 5) and found that the standardised factor loadings between the latent variables and corresponding observational variables were >0.5. We also conducted a hypothesis test (see Table 6 and Figure 4) and found that hypotheses H1, H2, H3, and H6 were valid, whereas hypotheses H4 and H5 were not. Based on these results, we revised the theoretical model by eliminating the paths that did not pass the hypothesis test, resulting in the revised model (see Figure 5).

**Table 5:** Results of the measurement model.

**Structure variables**	**Observation indicators**	**Standardised path coefficient**	***p*-value**
Commuting satisfaction	CS01	0.814	^***^
	CS02	0.795	^***^
	CS03	0.773	^***^
	CS04	0.831	^***^
	CS05	0.881	^***^
	CS06	0.873	^***^
Positive emotion	PE01	0.791	^***^
	PE02	0.788	^***^
	PE03	0.759	^***^
	PE04	0.711	^***^
	PE05	0.844	^***^
	PE06	0.831	^***^
	PE07	0.810	^***^
	PE08	0.823	^***^
	PE09	0.792	^***^
	PE10	0.769	^***^
	PE11	0.696	^***^
	PE12	0.669	^***^
	PE13	0.684	^***^
	PE14	0.722	^***^
Barrier-free environment perception	BEP01	0.705	^***^
	BEP02	0.765	^***^
	BEP03	0.770	^***^
	BEP04	0.907	^***^
	BEP05	0.909	^***^
	BEP06	0.816	^***^
Commuting attitude	CA01	0.726	^***^
	CA02	0.857	^***^
	CA03	0.796	^***^
	CA04	0.743	^***^
	CA05	0.577	^***^
	CA06	0.642	^***^
	CA07	0.637	^***^

^***^p ≤ 0.01.

**Table 6:** Results of the structural model.

**Hypothesis**	**Connexion**	**Standardised path coefficient**	***t-*value**	***p*-value**
H1	Barrier-free environment perception → commuting satisfaction	0.277	5.344	0.000
H2	Barrier-free environment perception → positive emotion	0.442	7.288	0.000
H3	Commuting attitude → positive emotion	0.376	6.212	0.000
H4	Commuting attitude → commuting satisfaction	−0.123	−0.695	0.487
H5	Commuting attitude → Barrier-free environment perception	0.166	1.628	0.104
H6	Positive emotion → commuting satisfaction	0.423	4.670	0.000

#### 5.4.3 Results of mediation effect testing

We used the bootstrap method to evaluate the mediation effect and estimated it based on a bias-corrected interval. If the 95% confidence interval (95% CI) for the indirect effect does not encompass zero, it indicates a statistically significant mediation effect. The model yielded direct, total, and indirect effects (see Table 7).

**Table 7:** Mediation effect results.

	**Mediation path**	**Indirect effect coefficient**	***p*-value (two-tailed)**	**95% CI**		**Estimate**
				**Lower**	**Upper**	
H7	Barrier-free environment perception - positive emotion - commuting satisfaction	0.145^***^	0.000	0.070	0.247	Valid
H8	Commuting attitude - positive emotion - commuting satisfaction	0.252^***^	0.001	0.118	0.426	Valid
	Commuting attitude - barrier-free environment perception - positive emotion	0.046^*^	0.102	−0.110	0.114	Invalid

^***^p ≤ 0.01, ^*^0.05 < p ≤ 0.1.

Based on the results of the mediation analysis, the CI for the “commuting attitude - barrier-free environment perception - positive emotion” mediating effect is (−0.110, 0.114), which includes zero, thus suggesting a non-significant mediation effect in this pathway. However, none of the CI values for “commuting attitude - positive emotion - commuting satisfaction” or “barrier-free environment perception - positive emotion - commuting satisfaction” include zero, indicating significant mediating effects in these paths. Moreover, the direct impact of commuting attitude on commuting satisfaction is observed to be statistically significant, highlighting that positive emotions fully mediate between commuting attitude and commuting satisfaction, while positive emotions partially mediate the connexion between barrier-free environment perception and commuting satisfaction.

## 6 Discussion and conclusion

We investigated the relationship between momentary perceptions of barrier-free campus environment stimuli and the commuting satisfaction of college students in the post-COVID-19 pandemic era using data from the new CSU campus. The results are expected to enhance our understanding of barrier-free campus environment perceptions and the level of satisfaction with college students' commuting experiences.

### 6.1 Influence of barrier-free environment perception on commuting satisfaction

It has been generally documented that a good perception of environmental features is associated with higher commuting satisfaction (29, 72). Consistent with this, we found that barrier-free environment perception was positively associated with commuting satisfaction (79). We propose a barrier-free environment as a built environment feature from the perspective of users' subjective evaluation. SEM was used to further explore the path relationships and mediating effects that exist between barrier-free environment perception, commuting satisfaction, positive emotions, and attitudes among college students. The effect of barrier-free environment perception on commuting satisfaction has a simple path of “barrier-free environment perception - commuting satisfaction” and a complex path of “barrier-free environment perception - positive emotion - commuting satisfaction” (80). The results of this study reveal that an individual's assessment of commuting and environmental satisfaction is mainly based on their perception of the barrier-free environment, physical activity, and social interaction during commuting. This includes direct satisfaction through the psycho-neurological pathway and indirect satisfaction through the corresponding emotional satisfaction pathway. A rich perception of a barrier-free environment may give students a positive psychological perception of a campus with perfect humanised construction. Positive psychology can stimulate individuals' emotional mechanisms and mobilise positive emotions to yield better commuting satisfaction.

Participating in educational, social, and extra-curricular activities on campus can have a positive impact on the emotional wellbeing of college students. In addition to this, easy physical access to these opportunities can further enhance the frequency and quality of these experiences. A barrier-free environment can also facilitate these educational and social encounters, making them more accessible to a wider range of students (20, 81). The internal roads, public spaces, and building facilities of a university campus together constitute the overall barrier-free environment of the campus. They allow for easy commuting and communication between different functional areas on campus. The planning and design of internal campus roads and public spaces is based on the campus's functional zoning, which helps realise the transportation grid (82). Proper planning can enhance the connectivity of various functional areas such as the teaching, college, and living areas. This, in turn, helps students move seamlessly through different areas. It is easier for students to cross paths with one another, thus increasing the possibility of collegiality and interdisciplinary dialogue and enhancing campus vitality (83). To support student wellbeing during a pandemic, academic institutions must create an inclusive and accessible campus environment where students are more likely to reach their full potential.

### 6.2 Influence of commuting attitude and positive emotion on commuting satisfaction

Based on the model path coefficients, subjective emotions have a greater direct effect on commuting satisfaction than environmental perceptions. Positive emotions have a significant positive effect on commuting satisfaction. College students' long-term positive emotions contribute to their subjective interactions and connexions with barrier-free environments. This leads to a higher sense of wellbeing in the environment and positively affects their instantaneous commuting satisfaction. In addition, long-term attitudes to COVID-19 do not have a significant effect on commuting satisfaction, which differs from most studies that have found that commuting-related attitudes and preferences have direct and indirect effects on commuting satisfaction (29, 78, 84). This difference may have occurred because since the COVID-19 pandemic has ended, the epidemic policy has changed. Some of the previously mentioned psychological factors that produce changes due to the risk of infection no longer pose a threat to commuting behaviours. The current study was conducted at only one university, where the student population may have more homogeneous commuting behaviours and no clear preference for prioritising commuting behaviour choices. Students' attitudes and preferences towards commuting may also have been affected by the limitations of the weather conditions in Changsha in May and June and the current status of transportation organisations on campus. Individual adaptation or aesthetic fatigue to the geographic environment may also explain this difference (28). Because attitudes and preferences are formed based on an individual's long-term experience, the campus commuting behaviour of the college student population may be a single journey day after day, and such repetitive journeys may affect students' attitudes and preferences related to campus commuting. Therefore, the effects of short- and long-term journeys on commuting satisfaction require further investigation. However, commuting attitudes indirectly affect commuting satisfaction through positive emotions, and the respondents' commuting attitudes had a significant positive effect on positive emotions. Respondents' preferred commuting behaviours imply better positive emotions, which in turn provide higher commuting satisfaction. This study shows that, in the post-COVID-19 pandemic era, attitudinal preferences for positive emotions towards commuting behaviours are focused on the importance of commuting efficiency (e.g. “Getting to my destination as soon as possible is important after the epidemic”, “Flexibility in commuting time is important after the epidemic”, “Predictability of commute time is important after the epidemic”, and “Getting to the destination by the cheapest mode of travel is important after the epidemic”).

Previous studies have explored the connexion between commuting satisfaction and all three individual demographic characteristics as well as psychological and behavioural factors (1, 28, 38, 45, 73). However, this study is the first to confirm the indirect and direct effects of long-term attitudes, positive emotions, and momentary environmental perceptions related to commuting on commute satisfaction. The results suggest that satisfaction assessment depends on perceptions based on momentary conditions related to places and people as well as long-term subjective attitudes based on experiences and perceptions. Overall, this study provides valuable insights for promoting humanised planning and the construction of campus commuting and barrier-free environments for college students in the post-COVID era.

## 7 Limitations and outlook

We acknowledge that this study has some limitations. First, the assessment of campus commuting satisfaction was based on individual differences, perceptions of environmental stimuli, and psychological and behavioural factors. However, it did not consider the effect of demographic characteristics on the perception of commuting satisfaction among public transportation users. Future studies should consider demographic characteristics in order to reveal the heterogeneity of perceptions. Second, the objectivity of the indicators may have been influenced by respondents' underestimation or overestimation of their psychological responses. This study also ignored the effects of active and passive travel on college students' psychological factors. Future studies should include physiological indicators to improve measurement accuracy. Finally, the study sample consisted only of college students, and future studies should expand the sample type and collect more field data from a wider range of public transportation routes.

## Data availability statement

The raw data supporting the conclusions of this article will be made available by the authors, without undue reservation.

## Ethics statement

Ethical review and approval was not required for the current study in accordance with the local legislation and institutional requirements. Written informed consent for participation was not required for this study in accordance with the national legislation and the institutional requirements.

## Author contributions

JL: Conceptualization, Resources, Supervision, Writing – original draft. CZ: Conceptualization, Formal analysis, Investigation, Methodology, Writing – original draft. XC: Conceptualization, Resources, Writing – review & editing. YP: Conceptualization, Methodology, Writing – review & editing. SL: Supervision, Writing – review & editing. WL: Formal analysis, Investigation, Resources, Writing – review & editing. YC: Formal analysis, Investigation, Writing – review & editing. YL: Formal analysis, Investigation, Writing – review & editing. LY: Writing – review & editing.
